# Identification of Host Factors Interacting with Movement Proteins of the 30K Family in *Nicotiana tabacum*

**DOI:** 10.3390/ijms252212251

**Published:** 2024-11-14

**Authors:** David Villar-Álvarez, Mikhail Oliveira Leastro, Vicente Pallas, Jesús Ángel Sánchez-Navarro

**Affiliations:** Instituto de Biología Molecular y Celular de Plantas (IBMCP), Universitat Politècnica de Valencia-CISC, 46022 Valencia, Spain; davilal@posgrado.upv.es (D.V.-Á.); molilea@ibmcp.upv.es (M.O.L.); vpallas@ibmcp.upv.es (V.P.)

**Keywords:** 30K movement proteins, host factors, AMV, TMV, CPMV, CaMV, CMV

## Abstract

The interaction of viral proteins with host factors represents a crucial aspect of the infection process in plants. In this work, we developed a strategy to identify host factors in *Nicotiana tabacum* that interact with movement proteins (MPs) of the 30K family, a group of viral proteins around 30 kDa related to the MP of tobacco mosaic virus, which enables virus movement between plant cells. Using the alfalfa mosaic virus (AMV) MP as a model, we incorporated tags into its coding sequence, without affecting its functionality, enabling the identification of 121 potential interactors through in vivo immunoprecipitation of the tagged MP. Further analysis of five selected candidates (histone 2B (H2B), actin, 14-3-3A protein, eukaryotic initiation factor 4A (elF4A), and a peroxidase-POX-) were conducted using bimolecular fluorescence complementation (BiFC). The interactions between these factors were also studied, revealing that some form part of protein complexes associated with AMV MP. Moreover, H2B, actin, 14-3-3, and eIF4A interacted with other MPs of the 30K family. This observation suggests that, beyond functional and structural features, 30K family MPs may share common interactors. Our results demonstrate that tagging 30K family MPs is an effective strategy to identify host factors associated with these proteins during viral infection.

## 1. Introduction

For a plant virus to establish an infection, it must multiply in the infected cell and move into adjacent cells to eventually invade the vasculature and reach the distal parts of the plant host. Cell-to-cell movement occurs through pores communicating plant cells called plasmodesmata (PD), while long-distance movement takes place in most cases through the phloem [1,2]. Over the course of evolution, viruses have developed different strategies to carry out viral movement. In most cases, these strategies involve the expression of one or more specialized proteins called movement proteins (MPs) [3].

MPs can play different roles during the viral movement. Due to their ability to interact with nucleic acids, these proteins can form ribonucleoprotein complexes (vRNPs) with the viral genome. Along with their association with elements of the cytoskeleton and the endoplasmic reticulum (ER), they facilitate the viral movement towards the cell periphery [3,4]. Once there, MPs assist the passage of the viral progenies into the adjacent cell by different mechanisms that involve the modification of either the size exclusion limit or the whole structure of the PD. Cell-to-cell movement can be executed in different viral forms: as viral particles, vRNPs, or virus replication complexes (VRCs) [2,5].

Plant viruses encode a wide diversity of MPs. Depending on the number of MPs they employ, RNA viruses can be categorized into four distinct groups. The first group consists of viruses that encode multiple MPs of varying size and structure. The second group, known as the triple gene block, is composed of RNA viruses encoding three MPs [6,7]. The third group of viruses encodes two small MPs and is referred to as the double gene block group [8]. Finally, many viruses belonging to numerous viral genera, move using a single MP: this is the case of the 30K family group [3,9,10].

The MPs of the members of the 30K family are characterized by a size of around 30 kDa and share biochemical and structural characteristics with the MPs of tobacco mosaic virus (TMV) [11,12]. Despite their shared characteristics, their primary structure is highly variable. A conserved LXDX50-70G motif is flanked by a variable N-terminus (Nt) and C-terminus (Ct). The secondary structure consists of four α-helices and seven β-elements. The Ct domain is highly unstructured. Its function is related to the interaction with the coat protein (CP), but it is not necessary for intercellular movement [13,14]. The analysis of its three-dimensional structure suggests the presence of a trypsin-resistant “central core” containing two hydrophobic regions [15]. Molecular analysis using the alfalfa mosaic virus (AMV) system has shown that 30K proteins from at least seven different viral genera, including both RNA and DNA viruses, are functionally interchangeable for local and systemic transport [13,16,17,18]. These results imply that 30K proteins have conserved secondary or tertiary structures that have the same basic functions in all of them [18,19].

MPs can have other functions beyond those strictly related to the viral movement. Through interaction with host factors, MPs are able to modify different cellular processes such as subcellular localization, the regulation of gene expression, signaling, immune response, or metabolism [20,21,22]. Thus, MP–host factor interactions can influence virus accumulation and pathogenesis in different ways [23]. When these interactions facilitate virus accumulation, the host factors involved are termed pro-viral. Conversely, if the interaction reduces or blocks virus propagation, the host factors are referred to as antiviral [24,25].

Studying the role that these interactions play in the viral cycle is essential to understanding the course of an infection and ultimately developing therapeutic targets. Most of the methods for identifying host factors that interact with MPs are based on the analysis of protein–protein interactions (PPIs) [21]. A wide variety of methods are available to accomplish such approaches. These methods are grouped into three categories: those developed using computational tools or in silico, those performed outside a living organism in vitro, and those performed inside a living organism in vivo. Of these, in vivo methods have the obvious advantage of preserving the native conditions under which the interaction occurs [26,27].

So far, yeast two-hybrid (Y2H) and co-immunoprecipitation (Co-IP) are among the most used methods for host factor identification in vivo [24,25,28]. The Y2H method has enabled the identification of many host factors. Nevertheless, Y2H can also produce a non-negligible number of false positives, and it requires the generation of gene libraries if no previous search has been made and forces proteins to share the same cellular space [29]. On the other hand, Co-IP is helpful for finding new host factors by sequencing the protein or proteins retained following the immunoprecipitation of a previously labeled protein [30]. The main constraint of this approach is the availability of the corresponding antisera to immunoprecipitate the protein of interest. This is particularly relevant in the case of MPs, which are recalcitrant in generating efficient antibodies. However, the use of tags that are translationally fused in-frame to the protein of interest has overcome this limitation and made more universal this technology.

In this work, we developed a method to identify host factors during a viral infection based on Co-IP. By understanding the structure of the 30K family MPs, we inserted a tag into the coding sequence of the AMV MP without altering its functionality. This approach allowed for immunoprecipitation and subsequent sequencing, through which we identified potential host factors that interact with the MP throughout the infection. Since immunoprecipitation was performed from infected tissue, the native conditions under which the interactions occurred were preserved. This strategy allowed us to identify the host factors that interact not only with AMV MP but also with the MPs of other viruses of the 30K superfamily, with DNA and RNA genomes and different intercellular movement mechanisms.

## 2. Results

### 2.1. The Introduction of Tags into the Coding Sequence of the AMV MP Alters but Does Not Prevent Intercellular Movement

**Figure 1:**
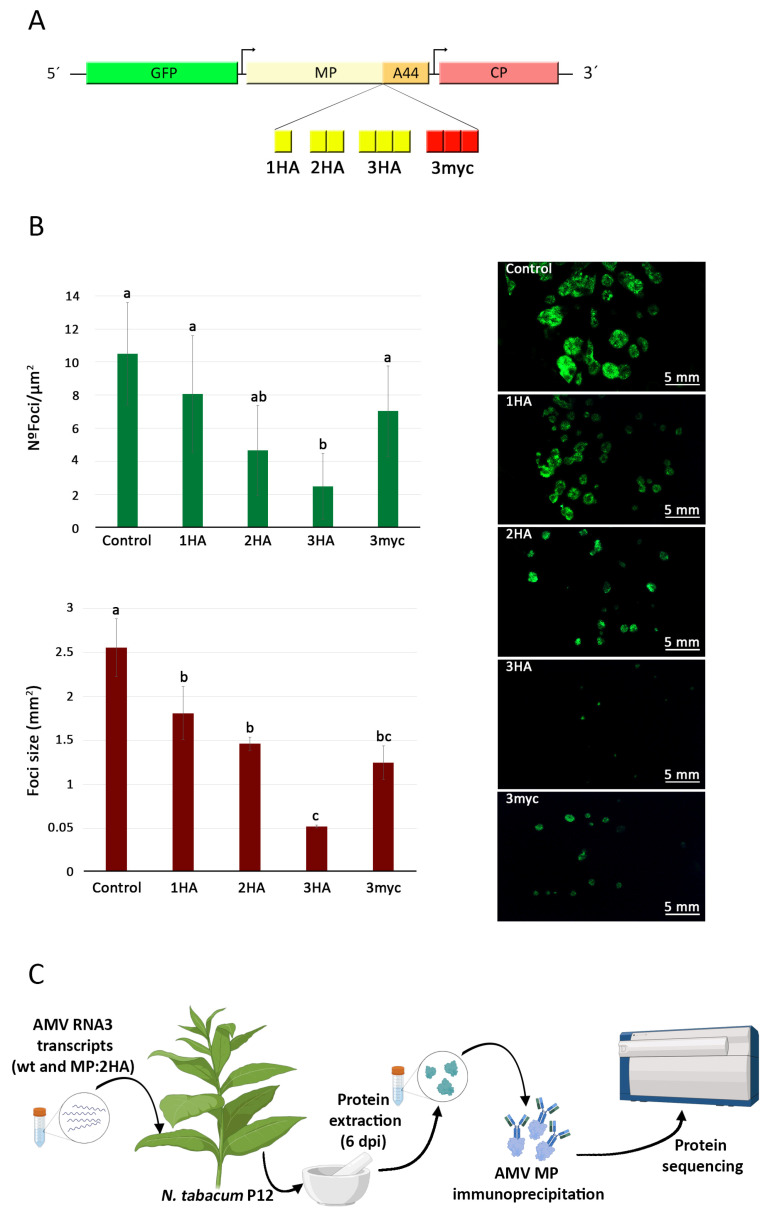
(**A**) Schematic representation of the AMV RNA 3 carrying the green fluorescence protein (GFP). Boxes correspond to the open reading frames of the GFP, the movement protein (MP), and the coat protein (CP), while arrows indicate the subgenomic promoter. The insertion site for the tags 1HA, 2HA, 3HA, or 3myc, between amino acids P256 and S257, is marked just before the C-terminal 44 amino acids of the MP (A44). (**B**) A quantitative analysis of the number (expressed as Nº foci/µm^2^) and size (expressed in mm^2^) of foci generated upon inoculation of transcripts corresponding to AMV cDNA3 wild type (control) or the constructs whose MPs carried 1HA, 2HA, 3HA, or 3myc epitopes is presented. The images on the right illustrate the infection foci generated by each of the aforementioned constructs. (**C**) Schematic representation of the procedure used to identify AMV MP interactors. *Nicotiana tabacum* p12 plants (plants constitutively overexpressing AMV replicase subunits 1 and 2) were inoculated with AMV cDNA3 transcripts carrying GFP at the 5′ end. Two versions of this cDNA3 were utilized: one with the MP bearing two HA tags and an untagged version, which served as a control. At six days post-inoculation, infected tissue was collected by visualizing the foci of infection. Subsequently, the total protein extraction and successive immunoprecipitation of AMV MP via the HA epitope were performed. The resulting immunoprecipitate was then subjected to mass spectrometry analysis. Experiments were performed in triplicate. Statistical analysis (Student’s *t*-test, *p* < 0.05) of the data enabled the identification of differences between groups, which are represented in the graph by the letters a, b, and c.

To identify host factors interacting with the AMV MP during the virus life cycle, two different epitope tags (hemagglutinin (HA) and human c-Myc protein (Myc)) were introduced into the coding sequence of the AMV MP. Tags were inserted between amino acids P256 and S257 (in isolate 425 Leiden, National Center for Biotechnology Information (NCBI); sequence number K03542.1). The insertion site was selected between the N-terminal functional transport domain and the C-terminal CP-interaction region [14]. A series of MP constructs with differing numbers of tags were tested. Three versions of the HA epitope were generated: one containing a single epitope (1HA), another with two epitopes (2HA), and the third with three epitopes (3HA). In the case of the myc epitope, a version of the MP with three myc epitopes repeated consecutively at the aforementioned position (3xmyc) was utilized, since the versions carrying one or two myc epitopes poorly reacted with the corresponding anti-myc antibody. To determine the impact of the tags in the MP-coding sequence on intercellular viral spread and the virus’s capability to disseminate the infection, a version of the AMV cDNA3 carrying the GFP gene at its 5′ end was employed [14], thereby enabling the infection to be tracked by detecting fluorescence in the infected cells (Figure 1A).

The different versions of AMV cDNA3, carrying the different epitopes in the MP plus a wild type (wt) construct, were inoculated into *Nicotiana tabacum* P12 plants (plants constitutively expressing the P1 and P2 subunits of the AMV polymerase) [31]. The inoculated leaves were observed under magnification at 3 days post-inoculation (dpi) to analyze both the number and size of infection foci generated with each version of cDNA3. Regarding the number of foci per µm^2^ (Nº foci/µm^2^), the cDNA3 versions carrying 1HA and 3xmyc in the MP did not show a significant decrease in comparison to the wt version. In contrast, the versions with 2HA and 3HA exhibited a more pronounced decrease of 56% and 76% in the number of foci per µm^2^, respectively, in comparison to the control (Figure 1B). The size of the foci, expressed in mm^2^, decreased in proportion to the number of HA epitopes present, with the versions containing 1HA, 2HA, and 3HA epitopes exhibiting a reduction of 30, 43, and 80%, respectively. The presence of three myc epitopes (3xmyc) resulted in a 51% decrease in mean foci size compared to the version without epitopes (Figure 1B). Based on these results, the AMV MP carrying two HA epitopes (2HA) was selected for the immunoprecipitation experiment and subsequent identification of potential host interactors.

### 2.2. Ontological Analysis of the Proteins Immunoprecipitated with the AMV MP

The Co-IP assay was conducted using the HA epitope and the Pierce Magnetic HA-Tag kit. Both versions of AMV cDNA3-GFP transcripts, namely the 2HA version and the untagged version, were inoculated onto *N. tabacum* P12 leaves by mechanical inoculation. Infection foci were collected at 6 dpi for protein extraction and subsequent MP-2HA immunoprecipitation (Figure 1C). The untagged version was employed as a control. Western blot analysis demonstrated the presence of AMV MP in both the crude protein extract and the final immunoprecipitate obtained from tissue inoculated with the 2HA version but not in those obtained from the tissue inoculated with the untagged version (Figure S1).

**Figure 2:**
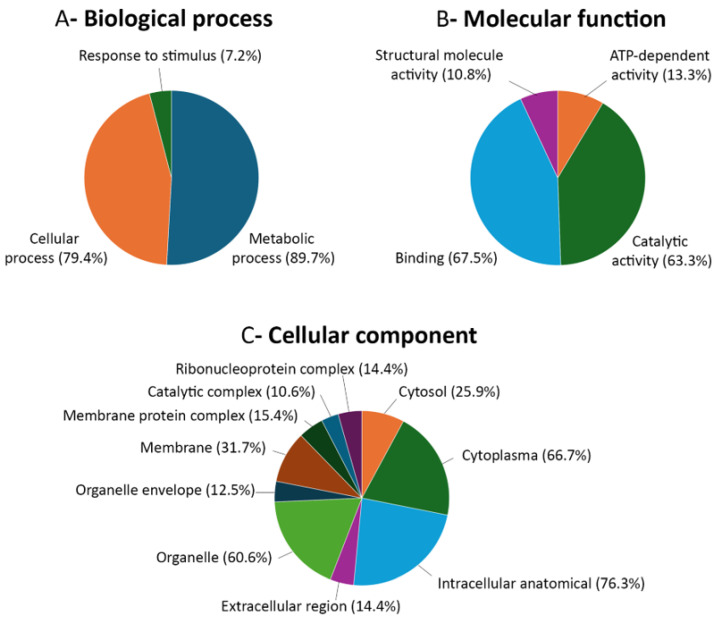
Ontological analysis of the proteins immunoprecipitated with the AMV MP. The graphs corresponding to the three ontological categories, (**A**) biological process, (**B**) molecular function, and (**C**) cellular component, are shown. Each graph illustrates the various subcategories into which the set of proteins under analysis was classified. The proportion of proteins in the sample assigned to each subcategory is indicated in brackets. Each protein can be assigned to more than one subcategory.

The immunoprecipitated proteins were subjected to mass spectrometry sequencing, resulting in the identification of a total of 121 potential interactors (see Table S1). Ontological analysis of these proteins was carried out using Omicsbox software (https://www.biobam.com/omicsbox, accessed on 17 July 2024), generating diagrams corresponding to the ontology divisions: biological process, molecular function, and cellular component. In terms of biological processes, the category to which the majority of proteins were assigned was metabolic processes, with 89.7% of the identified proteins. The second-largest category was that of cellular processes, with 79.4% of the proteins assigned to it. Approximately 7.1% of the proteins were assigned to the category of response to stimulus (Figure 2A). Concerning molecular function, 67.0% of the proteins were assigned to the binding category, 63.3% to catalytic activity, 13.3% to ATP-dependent activity, and 10.8% to structural molecule activity (Figure 2B). With regard to the cellular component, the three categories to which the highest number of proteins were assigned were intracellular anatomical (76.3%), cytoplasm (66.7%), and organelle (60.6%). The remaining categories, to which a smaller number of proteins were assigned, were membrane (31.7%), cytosol (25.9%), membrane protein complex (15.4%), ribonucleoprotein complex (14.4%), extracellular region (14.4%), organelle envelope (12.5%), and catalytic complex (10.6%) (Figure 2C).

### 2.3. Some Interactors Identified with the AMV MP Might Be Common in the MP 30K Family

**Figure 3:**
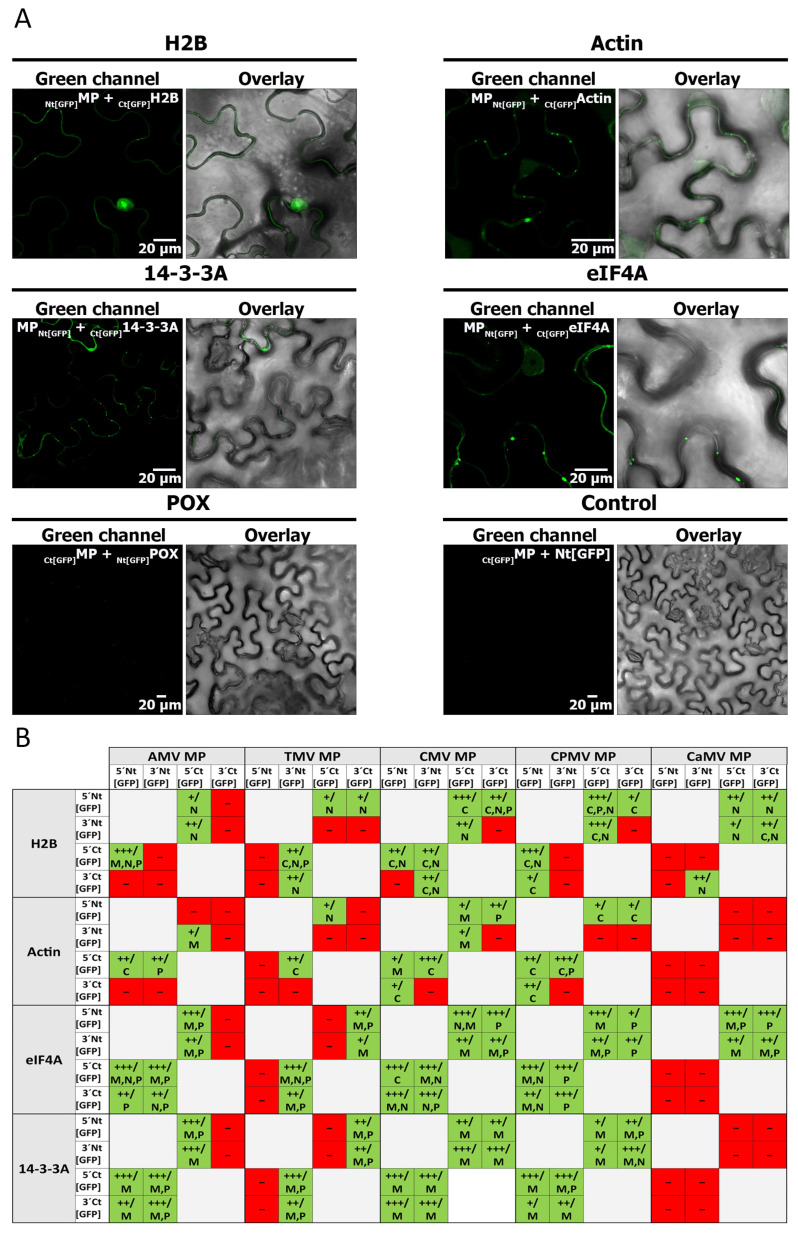
The bimolecular fluorescence complementation (BiFC) between MPs of AMV, TMV, CMV, CPMV, and CaMV and the selected interactors (histone 2B (H2B), actin (Act), 14-3-3A, eukaryotic initiation factor 4A (eIF4A), and peroxidase (POX)) is presented. (**A**) Confocal microscopy images of representative combinations of the analysis of each interaction with the AMV MP are shown. The scale bar represents 20 µm. (**B**) Table summarizing the results of the BiFC experiments on the interaction of the MPs of the five viruses with each of the proteins analyzed. The table shows all BiFC combinations in which the Nt or Ct fragments of the GFP are placed at the N-terminus (5′Nt [GFP], 5′Ct [GFP]), or the C-terminus (3′Nt [GFP], 3′Ct [GFP]) of the corresponding protein. The results are indicated as positive (+, green) or negative (−, red), and the intensity of the signal obtained under the same conditions (+, low intensity; ++, medium intensity; +++, high intensity) is noted. The most likely cellular location suggested by the images (C, cytoplasm; M, membrane; N, nucleus; P, plasmodesmata) is also indicated. The free Nt end of GFP was employed in the BiFC negative controls for all proteins analyzed.

To validate the interaction of the potential interactors with the AMV MP with a different method, five candidate proteins were selected based on the number of reads obtained with each of them in the sequencing of the eluted peptides and the potential biological significance of the interaction. The selected proteins were histone 2B (H2B, LOC107775690), actin (Act, LOC107831313), 14-3-3A (LOC107765116), eukaryotic initiation factor 4A (eIF4A, LOC107764101), and peroxidase (POX, LOC107808758). Bimolecular fluorescence complementation (BiFC) experiments were conducted between the AMV MP and the five candidate proteins. The reconstitution of the GFP fluorescence signal was visualized in all of them, except for POX (Figure 3A). The nuclear-localized GFP signal was observed in interactions between AMV MP with H2B and eIF4e (Figure 3A,B), as expected. Punctate structures at the cell periphery that resembled the plasmodesmata localization were visualized in all positive interactions: AMV MP-H2B, AMV MP-actin, AMV MP-14-3-3A, and AMV MP-eIF4e (Figure 3A,B).

To determine whether the interactors identified by the immunoprecipitation of AMV MP were shared by MPs of the 30K family, five more MPs of this family corresponding to tobacco mosaic virus (TMV), cucumber mosaic virus (CMV), cowpea mosaic virus (CPMV), and cauliflower mosaic virus (CaMV), which differ in both their genome and their type of intercellular movement, were also evaluated by BiFC. Similar to the AMV MP, these MPs showed a positive result for interaction with H2B and eIF4A proteins. MP interaction with 14-3-3A and actin proteins was positive for all MPs except the CaMV MP. However, no MP was found to interact with the POX protein (Figure 3 and Figure S2). Taken together, these results suggest that the MPs of the 30K MP family might share common interactors, regardless of how different viral properties are.

### 2.4. The Strategy Used to Identify Host Factors Interacting with the AMV MP Is Effective in Uncovering Components of a Multi-Protein Complex

**Figure 4:**
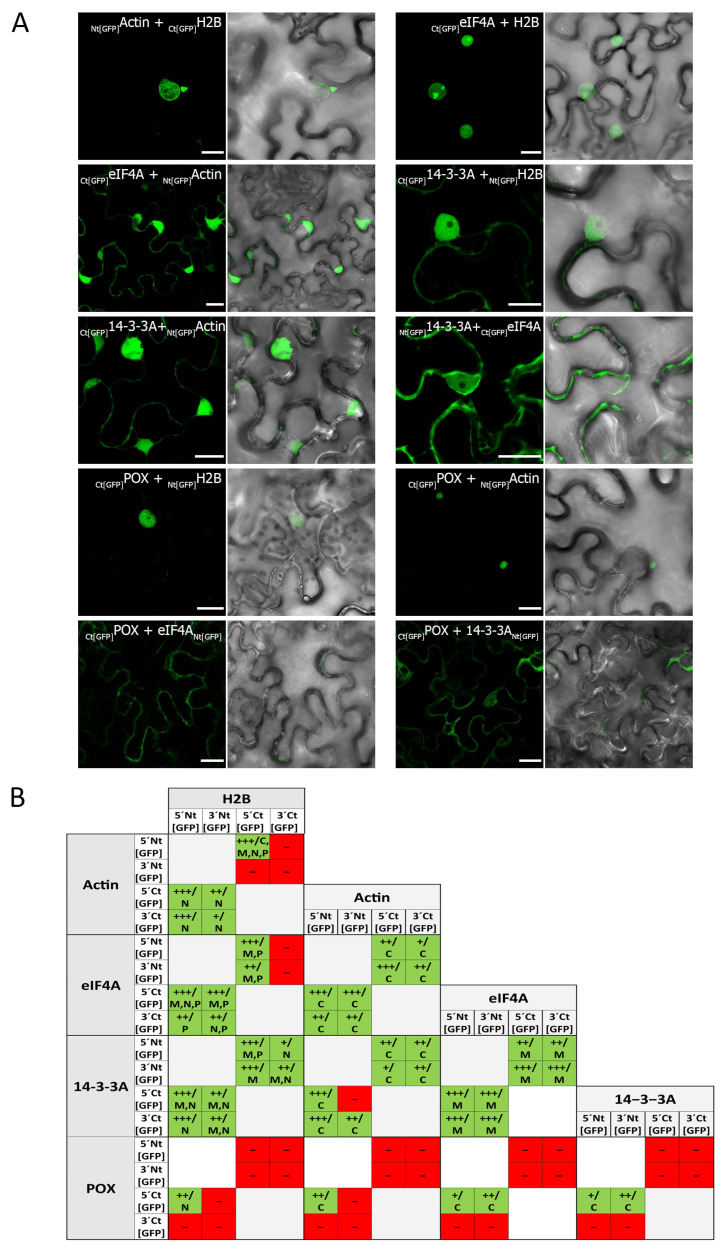
The bimolecular fluorescence complementation (BiFC) experiments conducted among all candidate interactors with each other (histone 2B [H2B], actin [Act], 14-3-3A, eukaryotic initiation factor 4A [eIF4A], and peroxidase [POX]). (**A**) Confocal microscopy images of representative combinations of the analysis of each interaction are shown. The scale bar represents 20 µm. (**B**) Table summarizing the results of the BiFC experiments conducted among all candidate interactors (H2B, Act, 14-3-3A, eIF4A, and POX). The table shows all BiFC combinations in which the Nt or Ct fragments of the GFP are placed at the N-terminus (5′Nt [GFP], 5′Ct [GFP]) or the C-terminus (3′Nt [GFP], 3′Ct [GFP]) of the corresponding protein. The results are indicated as positive (+, green) or negative (−, red), and the intensity of the signal obtained under the same conditions (+, low intensity; ++, medium intensity +++, high intensity) is noted. The most likely cellular location suggested by the images (C, cytoplasm; M, membrane; N, nucleus; P, plasmodesmata) is also indicated.

**Figure 5:**
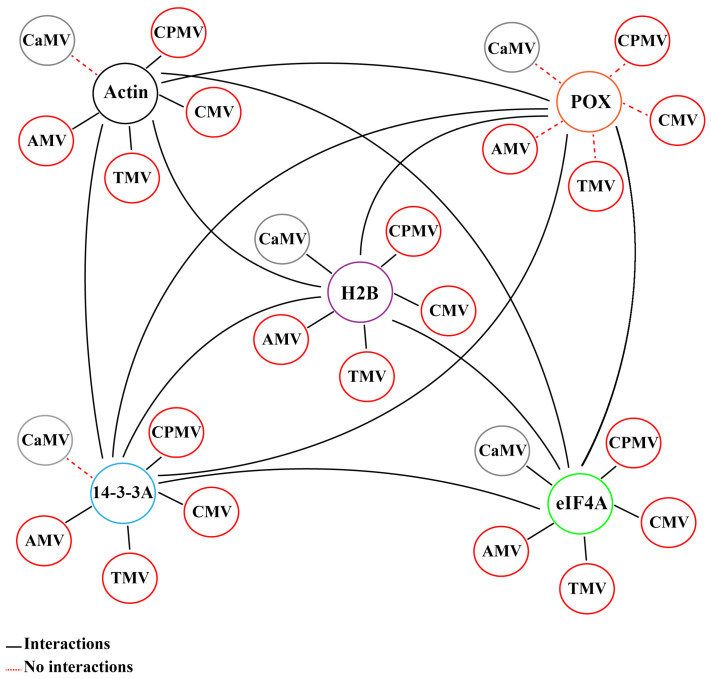
The proposed interactome is presented graphically, comprising the host factors studied with the five MP of the 30K family. The interactors are histone 2B (H2B), actin (Act), 14-3-3A, eukaryotic initiation factor 4A (eIF4A), and peroxidase (POX). MPs are identified by the name of the virus to which they belong: cauliflower mosaic virus (CaMV), cowpea mosaic virus (CPMV), cucumber mosaic virus (CMV), tobacco mosaic virus (TMV), and alfalfa mosaic virus (AMV). Solid and dashed lines between each component indicate positive and negative BiFC interaction, respectively.

BiFC experiments conducted with the AMV MP yielded positive results for the interaction of the AMV MP with four of the five candidate proteins tested. However, neither the AMV MP nor any of the 30K MP family tested yielded positive results for the interaction with the POX protein. One explanation for this result is that POX forms part of a multi-protein complex together with the other candidate proteins. To test whether the identified interactors were part of a protein complex, the interaction of the five selected interactors (H2B, Act, 14-3-3A, eIF4A, and POX) with each other was examined by BiFC. The results confirmed the interaction between all interactors. The fluorescence localization of the interactions was predominantly either nuclear or cytoplasmic. The localization at the plasma membrane or plasmodesmata was less frequent (Figure 4A,B). The interaction of the five interactors with each other suggests that the five protein candidates form part of a multi-protein complex and that POX was carried over during immunoprecipitation as part of this complex. In this hypothetical interactome, all the host proteins, with the exception of POX, interact with the AMV MP (Figure 5). Consequently, the results of this study indicate that the in vivo immunoprecipitation of AMV MP is an effective approach for the identification of interactors of the 30K MP family and for the identification of proteins that may form part of multi-protein complexes associated with them.

## 3. Discussion

Here, we show that the introduction of tags into the coding sequence of the AMV MP has proven to be an effective strategy for the identification of host factors by immunoprecipitation of the viral protein during the course of infection. This is relevant since most of the studies dealing with the interactome of viral proteins have been carried out by overexpressing the protein of interest but not in the context of a viral infection. This approach has led to the identification of 121 potential candidates. Five of the potential candidates (H2B, Act, 14-3-3A, eIF4A, and POX) were selected based on both statistical and biological considerations. Their corresponding interactions were subsequently analyzed by an alternative method. Of the five candidates, four were confirmed to directly interact with the AMV MP, while POX was proven to interact with the other four candidates but not with the viral protein, suggesting that it forms part of a multi-protein complex related to the AMV MP.

In addition to immunoprecipitation, one of the most employed host factor identification approaches is the yeast-two hybrid (Y2H) system [32]. Zuidmeer-Jongejan (2002) utilized this system to identify host factors related to AMV MP [33]; however, the results were inconclusive. Subsequently, in order to overcome the limitations associated with the hydrophobic domain of the protein within the Y2H system, the same approach was carried out using a version of the MP that lacked the hydrophobic domain. The truncated version enabled the identification of patellins 3 and 6 as AMV MP interactors [15]. In contrast to the Y2H system used by Peiró et al. (2014) [15], the strategy presented in this work has the following advantages: (i) it allows for the use of a complete and functional MP; (ii) it is not necessary to create a protein library; and (iii) it allows for the immunoprecipitation of host factors during the course of infection.

The ontological analysis of the 121 candidates revealed that they exhibited variability within the three ontological categories. Of particular interest are the results obtained in the cellular component category, as they identify host factors associated with different cellular structures, including organelles, membranes, and cellular anatomical structures. The subcellular localization of the five candidates selected reflects this variability. H2B has a nuclear localization [34], whereas the 14-3-3 and eIF4A proteins manifest a variable subcellular localization, which can extend to the nucleus and cytoplasm [35,36,37]. POX proteins have been identified in several subcellular locations, including the cytosol, stroma, thylakoid membrane, and cell wall [38,39]. Actin, on its part, is primarily associated with the cytoskeleton [40]. The extensive range of locations of these proteins indicates that the system employed is applicable for the identification of interactors associated with any subcellular compartment.

The five selected proteins also display considerable functional diversity. Their potential roles in the viral cycle, which may be influenced by their interactions with MPs, are therefore diverse. The H2B protein has been assigned biological functions that extend beyond its structural role. For example, the post-translational modification of this protein has been linked to the regulation of the genetic response to different types of stress, including that generated by viral infections [41]. An illustrative example of the potential role of H2B in the plant defensive response to RNA viruses is provided by the study of potato virus X (PVX) in Nicotiana benthamiana. The repression of the H2B gene was found to result in a reduction in PVX accumulation, which was attributed to an SA-mediated response [42].

Actin is a crucial protein in the formation of the cytoskeleton in eukaryotic cells. The actin cytoskeleton is involved in numerous cellular processes and is a key player in pathogenesis [43,44]. For instance, Act plays a significant role in the intracellular transport of viral proteins and ribonucleoprotein complexes during infection [45]. Previous studies have already demonstrated that TMV and CMV MPs used actin filaments for intracellular movement [46,47,48]. The present work has demonstrated that the MPs of these two viruses, as well as those of AMV and CPMV, bind Act. This suggests that viral MPs that use actin microfilaments for intracellular movement may bind Act in addition to other actin-associated proteins.

Notably, 14-3-3 proteins serve as signal transducers in response to a multitude of stimuli. The transduction process is primarily initiated through the binding of these proteins to phosphorylated proteins. Consequently, 14-3-3 proteins are implicated in a plethora of cellular processes, including metabolism, hormone signaling, and stress responses. Furthermore, these proteins play a significant role in the defensive responses to a variety of pathogens, including viruses [49,50]. For instance, the *14-3-3* gene is upregulated in response to TMV infection, while the 14-3-3 protein interacts with the helicase domain of the TMV replicase [51]. Another illustration of the significance of these proteins in cellular responses to viral infections was provided by the beet black scorch virus (BBSV). The CP of this virus targets 14-3-3a to subvert mitogen-activated protein kinase (MAPKKKα)-mediated antiviral immunity in plants [52].

eIF4A is a member of the RNA helicase protein family carrying the amino acid sequence D-E-A-D (asp-glu-ala-asp) or DEAD-box. The primary function of this group of proteins is to facilitate the unfolding of mRNA, which is necessary for ribosome binding during translation initiation. In addition, eIF4 proteins are involved in the non-canonical translation of viral RNAs. The interaction between these factors and viral RNA has been extensively documented, and they are considered attractive targets for the success of antiviral strategies [53].

Finally, POX proteins are involved in cellular responses to different stimuli, catalyzing the oxidation of various organic compounds. In response to biotic stress, they are implicated in the production of H_2_O_2_, which triggers the activation of pathogen-responsive genes [54]. The expression of POX genes can change as a response to pathogen stress, as it occurs during TMV infection [55] or in apricot seeds infected with prunus necrotic ringspot virus, a virus closely related to AMV [56].

In light of the aforementioned information, it can be concluded that the method presented in this work is effective in identifying host factors relevant to the development of AMV viral infection. It is also important to consider that some of the identified interactors of AMV MP are also interactors of other MPs of the 30K family. The MP 30K family of viruses subjected to this study exhibited a variety of distinct characteristics, including differences in the type of intercellular movement and genome composition. The genomes of AMV, TMV, CMV, and CPMV are composed of RNA, whereas that of CaMV is composed of DNA. All of the aforementioned viruses form tubular structures for intercellular movement, with the exception of TMV. CaMV and CPMV move in the form of viral particles, whereas TMV moves in the form of ribonucleoprotein complexes (vRNPs). AMV and CMV are capable of moving either in the form of vRNPs or viral particles [3,5,12]. The observation that 30K MPs are functionally interchangeable in the AMV system [16] suggests shared functional characteristics despite the great diversity among these viruses. This could explain why they also might share common interactors. Further investigation is required to ascertain the precise role of each of these factors in AMV infection and other viruses within the 30K MPs. Nevertheless, the discovery of common factors interacting with multiple members of the 30K MP family members opens exciting research lines involving the characterization of how these viruses are translocated within the plant.

## 4. Materials and Methods

### 4.1. DNA Manipulation

#### 4.1.1. Constructions for Bimolecular Fluorescence Complementation (BiFC) and Competitive Binding Assays

We performed the BiFC assay with the constructions pSK35S-NtGFP:-PoPit, pSK35S-CtGFP:-PoPit, pSK35S-:NtGFP-PoPit, and pSK35S-:CtGFP-PoPit, which permitted N- and C-terminal fusion of the enhanced green fluorescent protein (EGFP) fragments (NtGFP and CtGFP) at the N- or C-terminus of a specific assayed protein by ligation with the NcoI/NheI restriction sites. Detailed handling for obtaining these plasmids was previously described [57]. For the analysis of in vivo interactions, the 30K MPs from AMV (AAA46295.1); TMV (NP_597748); CPMV (CAA25315.1); CMV (BAA01396.1); CaMV (CAA23456.1); *N. tabacum* H2B-like (LOC107775690, hereinafter referred to as H2B only); actin (LOC107831313); peroxidase (LOC107808758); eIF4A (LOC107764101); and 14-3-3A (LOC107765116) were fused with the pSK plasmids described above to obtain the 4 possible combinations. The expression cassettes were subcloned into the pMOG800 binary vector. All genes were amplified using gene-specific oligonucleotides targeting the 5′ and 3′ ends of each gene. All DNA manipulations were confirmed by plasmid DNA sequencing.

#### 4.1.2. Constructions for Immunoprecipitation Assays

For in vivo co-immunoprecipitation assays, HA or Myc tags were introduced into the coding sequence of the AMV MP. For this purpose, the AMV cDNA 3 clone (pGFP/A255/CP) [14], which expresses the GFP, was modified by replacing the wt version of the MP with the HA or Myc tagged version using the NcoI/NheI restriction sites. Epitopes were introduced between amino acids 256 and 257 of the AMV MP by PCR amplification using specific oligonucleotides containing the epitope sequence.

### 4.2. Inoculation of P12 Plants and Infection Foci Analysis

To analyze cell-to-cell movement, the AMV cDNA3 constructs were amplified from the corresponding plasmid using specific pair primers targeting the 5′ and 3′ termini of the viral sequence, with the sense primer containing the T7 promoter sequence. The generated amplicons were then used directly as templates for in vitro transcription with T7 RNA polymerase (Takara Bio Inc., Shiga, Japan). Transgenic *Nicotiana tabacum* P12 plants, which express the polymerase proteins P1 and P2 of AMV [31] were grown under long-day photoperiods of 16 h light at 25 °C and 8 h dark at 22 °C and inoculated by gently rubbing transcripts onto leaves. The infected areas on P12 plants were photographed using a Lupe microscope (MZZ16F Leica), with measurements taken at 3 dpi using ImageJ software version 2.0r. Graphs depict the average size in mm2 of the infection foci. Briefly, 10 µL of the transcription mixture per leaf was used to inoculate each construct.

### 4.3. Agrobacterium tumefaciens-Mediated Transient Expression and BiFC Assays

In transient expression assays conducted in *N. benthamiana*, the binary vectors acquired previously were introduced into *Agrobacterium tumefaciens* strain C58C1 by electroporation. The transformed bacteria were then cultured overnight in a liquid Luria–Bertani (LB) medium supplemented with kanamycin (50 mg/mL^−1^) and rifampicin (100 mg/mL^−1^) antibiotics for agrobacterium and plasmid selection. Subsequently, the bacterial cultures were centrifuged and resuspended to achieve the desired final OD600 value of 0.4 using a solution composed of 10 mM MgCl_2_ and 10 mM MES adjusted to pH 5.6. These suspensions were gently infiltrated into the abaxial side of two-week-old *N. benthamiana* leaves by applying slight pressure. For experiments involving BiFC, which require the simultaneous expression of two different proteins, bacterial cultures were adjusted to an OD600 value of 0.4 and then mixed in equal proportions before infiltration. The plants were cultivated under long-day photoperiods, consisting of 16 h of light at 25 °C and 8 h of darkness at 22 °C.

### 4.4. Laser Scanning Confocal Microscopy and Image Analysis

The BiFC and translocation experiments were observed using an inverted Zeiss LSM 780 laser scanning confocal microscope (Carl Zeiss in Oberkochen, Germany). BiFC assays were observed two days after the agroinfiltration of *N. benthamiana* leaves. Leaves were observed under the confocal microscope at 3 dpi. Excitation for eGFP was achieved with lasers emitting light at wavelengths of 488 nm. Emission detection windows were set between 492 and 532 nm. Chlorophyll excitation utilized a 488 nm wavelength, and emission data were collected above 700 nm. Image processing and analysis, including overlays and Z-stack projections, were carried out using the FIJI software (version 2.9.0) [58].

### 4.5. Co-Immunoprecipitation Assay (Co-IP)

The Co-IP assay was conducted using the HA epitope and the Pierce Magnetic HA-Tag kit (Thermo Fisher Scientific, Waltham, MA USA), following the manufacturer’s guidelines. The AMV cDNA3-GFP construct transcripts, comprising the HA epitope within the MP coding sequence, were mechanically inoculated onto *N. tabacum* P12 leaves through gentle rubbing with carborundum. A non-epitope version of cDNA3-GFP was used as the control. Infected tissue weighing 60 mg was obtained at 6 dpi by visualizing GFP under magnification to select infection sites. The tissue was homogenized with liquid nitrogen and 0.5 mL of denaturing buffer (50 mM Tris HCl, pH 8.0; 150 mM NaCl; 1% NP-40; and 0.5% sodium deoxycholate; and 0.1% SDS). The extracts were incubated by constant agitation for 2 h at 4 °C. Briefly, 40 µL of the supernatant was used for the protein expression control, while the remaining supernatant was incubated with 20 µL (0.2 mg) of Pierce Anti-HA Magnetic Beads for 90 min at room temperature with agitation. Following washing steps with non-denaturing buffer (25 mM Tris HCl, pH 7.5; 150 mM NaCl; 1 mM EDTA; 1% NP-40; and 5% glycerol), the HA-immunoprecipitated proteins were eluted by resuspending the beads with 100 µL of 1× Laemmli buffer and incubated at 100 °C for 10 min. Western blot analysis was conducted using 20 µL of the expression control, 30 µL of IP proteins per line, and a monoclonal anti-HA antibody (Sigma-Aldrich, Steinheim, Germany), in accordance with the manufacturer’s instructions. The rest of the IP eluate was sent for mass spectrometry sequencing at the proteomic service of the University of Córdoba. Functional annotation and analysis were conducted using OmicsBox (https://www.blast2go.com/, accessed on 17 July 2024) by using its default settings, followed by Gene Ontology mapping, where each potential GO term was given a GO annotation score.

## Data Availability

All the experimental data related to this work are included in the manuscript, figures, or Supporting Information.

## Supplementary Materials

The following supporting information can be downloaded at: https://www.mdpi.com/article/10.3390/ijms252212251/s1.
