# Supplementary material for: Identification of Host Factors Interacting with Movement Proteins of the 30K Family in Nicotiana tabacum
Source: Int J Mol Sci. 2024 Nov 14;25(22):12251. doi: 10.3390/ijms252212251 (PMC11595209; doi:10.3390/ijms252212251)
Supplement: Supplementary file 1 [file ijms-25-12251-s001.zip › Figure S1.pptx]

## Slide 1
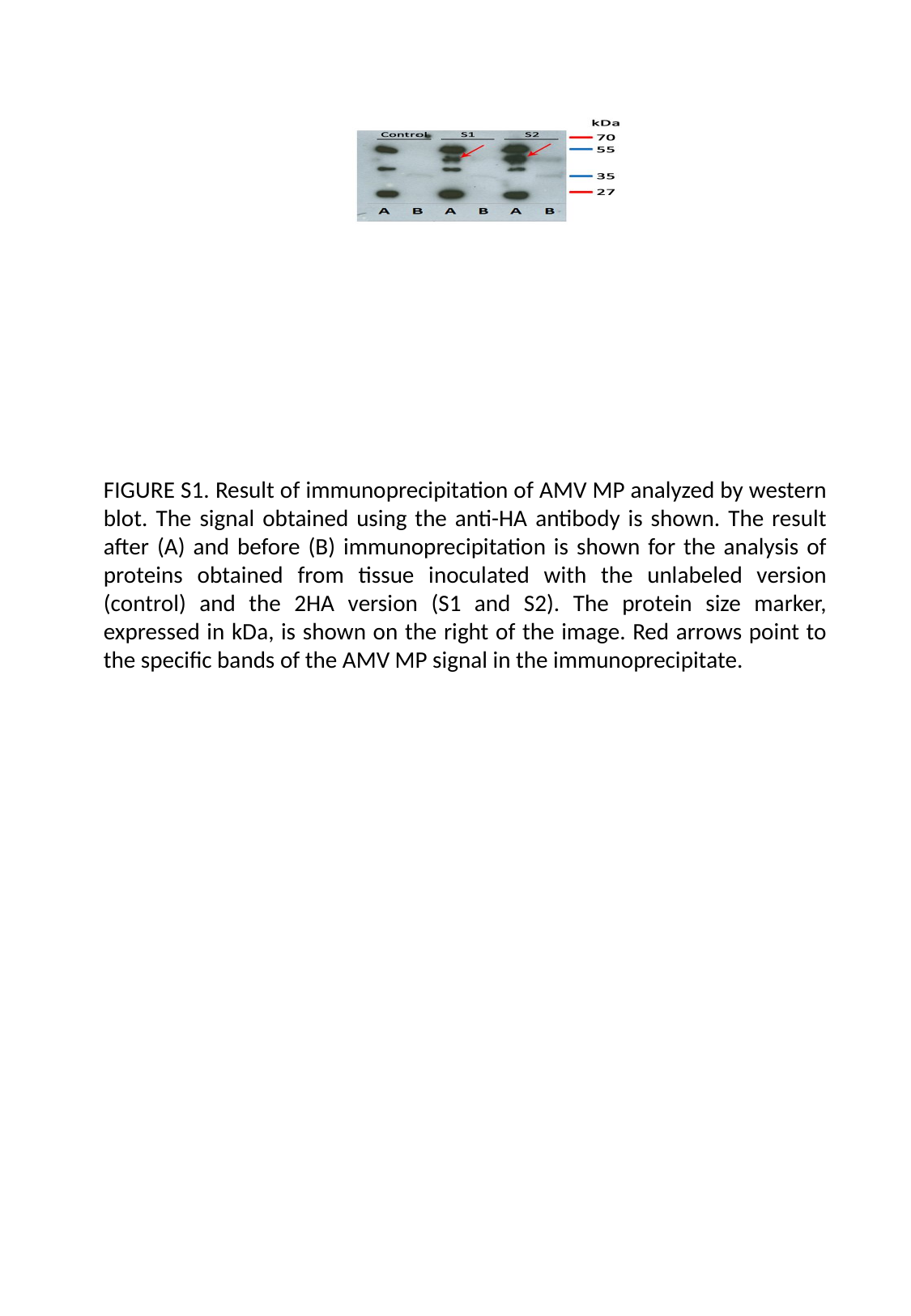

FIGURE S1. Result of immunoprecipitation of AMV MP analyzed by western blot. The signal obtained using the anti-HA antibody is shown. The result after (A) and before (B) immunoprecipitation is shown for the analysis of proteins obtained from tissue inoculated with the unlabeled version (control) and the 2HA version (S1 and S2). The protein size marker, expressed in kDa, is shown on the right of the image. Red arrows point to the specific bands of the AMV MP signal in the immunoprecipitate.
