# Supplementary material for: Identification of Host Factors Interacting with Movement Proteins of the 30K Family in Nicotiana tabacum
Source: Int J Mol Sci. 2024 Nov 14;25(22):12251. doi: 10.3390/ijms252212251 (PMC11595209; doi:10.3390/ijms252212251)
Supplement: Supplementary file 1 [file ijms-25-12251-s001.zip › Figure S2.pptx]

## Slide 1
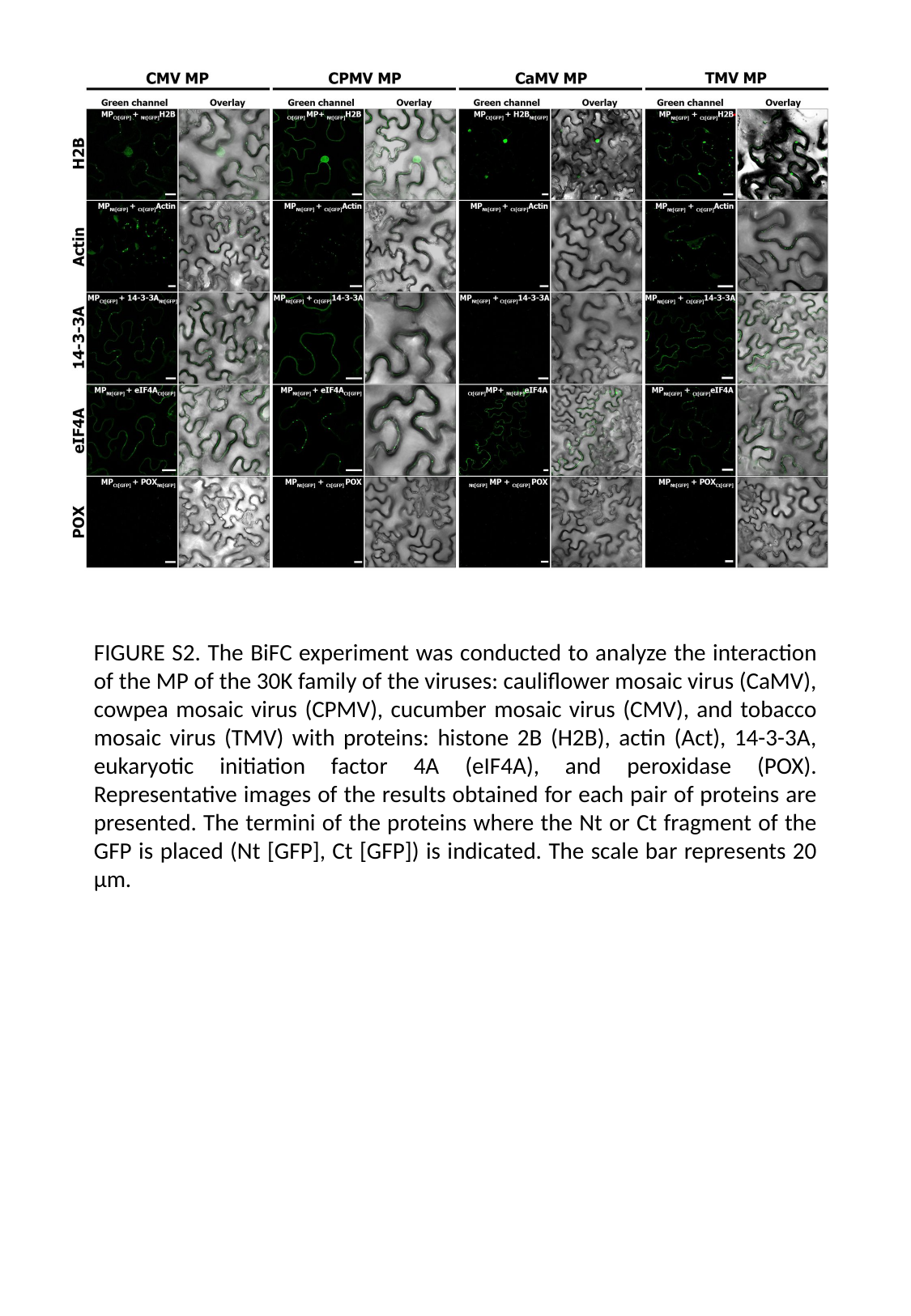

FIGURE S2. The BiFC experiment was conducted to analyze the interaction of the MP of the 30K family of the viruses: cauliflower mosaic virus (CaMV), cowpea mosaic virus (CPMV), cucumber mosaic virus (CMV), and tobacco mosaic virus (TMV) with proteins: histone 2B (H2B), actin (Act), 14-3-3A, eukaryotic initiation factor 4A (eIF4A), and peroxidase (POX). Representative images of the results obtained for each pair of proteins are presented. The termini of the proteins where the Nt or Ct fragment of the GFP is placed (Nt [GFP], Ct [GFP]) is indicated. The scale bar represents 20 µm.
